# Primary Intraosseous Vascular Malformation in a Child with *ELMO2* Mutation: Diagnostic and Dental Management Challenges

**DOI:** 10.3390/dj13100473

**Published:** 2025-10-16

**Authors:** Nadezhda Mitova, Valentina Petkova-Ninova, Peter Bakardjiev

**Affiliations:** Department of Pediatric Dentistry, Faculty of Dental Medicine, Medical University-Sofia, 1431 Sofia, Bulgaria; v.petkova@fdm.mu-sofia.bg (V.P.-N.); bakardjiev@fdm.mu-sofia.bg (P.B.)

**Keywords:** vascular malformation, intraosseous lesion, *ELMO2* mutation, pediatric dentistry, craniofacial anomalies

## Abstract

**Background/Objectives:** Vascular Malformation—Osteolytic Subtype (VMOS) is an exceptionally rare autosomal recessive disorder caused by homozygous pathogenic variants in the *ELMO2* gene, with fewer than ten genetically confirmed pediatric cases reported worldwide. This report presents the longitudinal dental management and clinical course of a child with VMOS, emphasizing the challenges of preventive and restorative care in such cases. **Methods:** A four-year-old child with a confirmed diagnosis of VMOS and a history of urgent bilateral coil embolization and surgical excision of mandibular aneurysmal bone cysts presented for dental care. The patient was followed for three years (2022–2025). Management focused on staged oral rehabilitation, preventive strategies, and restorative interventions adapted to changes across dentition stages. **Results:** At initial presentation, the child exhibited mandibular swelling, gingival hypertrophy, and a history of spontaneous intraoral bleeding. The postoperative course had been complicated by cerebral abscesses requiring prolonged intravenous antibiotics. During the primary dentition stage, full oral rehabilitation and strict preventive protocols were implemented to minimize caries and infection risk. In the mixed dentition period, the permanent incisors and molars erupted with enamel hypoplasia and developmental defects, necessitating composite restorations. Ectopic eruption and suboptimal oral hygiene, partly related to parental fear of bleeding, were also managed with reinforced preventive counseling. **Conclusions:** This case highlights the long-term dental implications of VMOS, underscoring the crucial role of the pediatric dentist in early preventive planning and individualized restorative management. Effective multidisciplinary coordination remains essential to preserve oral health and minimize complications in rare vascular syndromes with craniofacial involvement.

## 1. Introduction

Vascular malformations of the craniofacial skeleton are rare congenital anomalies characterized by the abnormal development of blood vessels within bone. Among these, the primary intraosseous subtype (VMOS) is particularly complex, presenting significant diagnostic and therapeutic challenges, especially in pediatric patients.

VMOS is a recently described autosomal recessive disorder caused by homozygous loss-of-function mutations in the *ELMO2* gene. These mutations result in fragile, immature vascular structures within bone. To date, fewer than ten genetically confirmed pediatric cases have been reported worldwide [1].

The *ELMO2* gene encodes a cytoskeletal adaptor protein essential for Rac1-mediated actin remodeling, cell migration, and apoptotic clearance. Loss of *ELMO2* function disrupts vascular smooth muscle cell adhesion and organization, leading to unstable vessel walls that are susceptible to arteriovenous malformations and aneurysmal changes [2,3]. Animal models with *ELMO2* inactivation exhibit severe vascular anomalies in the pharyngeal arch arteries, closely mirroring the human phenotype [1].

VMOS remains an exceedingly rare autosomal recessive condition, with fewer than ten genetically confirmed pediatric cases reported worldwide. Clinical manifestations typically emerge after 18 months of age and may progress insidiously, with expanding intraosseous lesions exerting pressure on developing dentition, orbital structures, or cranial nerves. Hemorrhagic episodes, whether spontaneous or iatrogenic, are frequent and may result in significant morbidity, including anemia or life-threatening complications. At present, no curative treatment exists for VMOS. Management of the condition in pediatric patients generally emphasizes early recognition, infection prevention, minimally invasive interventions, and long-term interdisciplinary follow-up [1,3,4,5,6].

Clinically, in children, VMOS presents with progressive jaw swelling, gingival hypertrophy, and recurrent spontaneous intraoral bleeding. Lesions most commonly affect the mandible and maxilla, intramembranous bones particularly susceptible to vascular malformations. Suspicion may be raised by the presence of radiolucent areas on imaging, mucosal hemangiomatous changes, premature tooth loss, or early tooth mobility [3,4]. Dental professionals are often the first to observe these early signs, highlighting their pivotal role in timely diagnosis.

Due to the high risk of hemorrhage, even minor procedures such as tooth extractions or biopsies can trigger life-threatening bleeding. In addition, oral infections may result in severe complications, including cerebral abscesses. Early diagnosis, supported by imaging and genetic testing, is essential for implementing preventive care and avoiding unnecessary surgical interventions [5,6].

Recent pediatric studies on craniofacial vascular malformations have documented long-term dental sequelae, including enamel hypoplasia, delayed tooth eruption, gingival hypertrophy, and increased susceptibility to caries. These findings emphasize the critical role of pediatric dentists in early detection, preventive care, and coordination of multidisciplinary management to mitigate potential complications [7,8,9]. By situating the present case within this context, we highlight the importance of structured dental monitoring and individualized care in rare intraosseous vascular disorders.

Multidisciplinary collaboration involving pediatric dentistry, oral surgery, radiology, genetics, and neurology is essential for optimal long-term care. Management should emphasize conservative rather than reactive strategies to minimize risk and preserve oral function [10].

This report presents the case of a boy initially evaluated at age four with gingival hypertrophy, spontaneous bleeding, and mandibular swelling. Genetic testing confirmed VMOS caused by a homozygous *ELMO2* mutation. His clinical course included arteriovenous malformations, emergency coil embolization, and secondary cerebral abscesses. Now nearly seven years old, he exhibits lasting dental sequelae, such as enamel hypoplasia of the permanent incisors. This case highlights the need for early recognition in pediatric dental settings and provides insights into managing rare vascular disorders with oral involvement.

## 2. Case Presentation

### 2.1. Patient Background

D.N. is a 7-year-old boy, born in Sofia, Bulgaria. During his early childhood, he lived in Cologne, Germany, but now resides with his family in Sofia. He was referred to the Faculty of Dental Medicine, Medical University of Sofia, by his father due to intraoral swelling and recurrent oral bleeding. At the initial examination in 2022, the patient was cooperative, with normal neurodevelopment. He exhibited characteristic craniofacial features, including prominent cheeks, a relatively large mandible, and hypertrophic zygomatic bones.

### 2.2. Medical and Genetic History

The patient was born at 37 weeks and 5 days of gestation via cesarean section due to fetal tachycardia detected on cardiotocography. His birth weight was 3300 g, and he measured 51 cm long. The pregnancy was complicated by gestational diabetes, managed with dietary modifications. There was no reported exposure to medications, tobacco, alcohol, or illicit substances during pregnancy. Prenatal imaging revealed mild polyhydramnios and an atrial septal defect in the third trimester. After birth, the patient exhibited mild respiratory distress and diastasis recti.

Genetic analysis confirmed a homozygous pathogenic variant in the *ELMO2* gene, consistent with a diagnosis of VMOS (Vascular Malformation—Primary Intraosseous Subtype). Both parents were heterozygous careers. The patient is the second child of a consanguineous union (third cousins). The mother experienced two prior stillbirths, with both fetuses demonstrating cerebral cysts and congenital heart defects. A younger sibling was also identified as an *ELMO2* mutation carrier. This family history highlights the critical role of genetic counseling in consanguineous families with recurrent fetal loss and suspected autosomal recessive vascular anomalies.

Prior to referral for pediatric dental evaluation, the patient underwent a comprehensive multidisciplinary work-up, including detailed family and medical history, and consultations with a pediatrician, pediatric neurologist, clinical geneticist, and immunologist. Genetic testing was performed on DNA extracted from EDTA-stabilized blood samples. Targeted enrichment of coding regions and exon–intron boundaries was achieved using the Agilent SureSelect Human All Exon V7 panel (Santa Clara, CA, USA), followed by next-generation sequencing (NGS) on the Illumina NextSeq 500 platform (San Diego, CA, USA). Variants meeting quality thresholds were confirmed via Sanger sequencing, including regions with low coverage or high homology.

Neuroimaging studies included computed tomography (CT), CT angiography, and magnetic resonance imaging (MRI) of the head. Neurodevelopmental assessment was conducted using the Denver II screening test. A prenatal ultrasound of the patient’s older sibling had revealed an atrial septal defect and a patent foramen ovale, with postnatal findings of diastasis recti. In the index patient, echocardiography confirmed the presence of a patent foramen ovale.

### 2.3. Course of Disease

Initial suspicion of VMOS was raised due to gingival hypertrophy and recurrent oral bleeding during the eruption of primary molars. Computed tomography angiography (CT-A) revealed an arteriovenous (AV) malformation located within the mandibular bone. During a scheduled biopsy under general anesthesia, the patient developed severe intraoperative hemorrhage, necessitating emergency bilateral coil embolization. Surgical management included excision of the vascular lesion and resection of an associated aneurysmal bone cyst on the left mandibular side, performed in 2019.

Intraosseous vascular malformations in VMOS pose unique clinical challenges due to their deep anatomical localization, high hemorrhagic potential, and the technical difficulty of achieving adequate hemostasis without compromising surrounding osseous structures. In this case, the postoperative course was further complicated by the development of three cerebral abscesses. These required prolonged hospitalization and intravenous antimicrobial therapy, including ampicillin, vancomycin, clindamycin, and metronidazole. Microbiological cultures isolate *Streptococcus intermedius*, *Staphylococcus epidermidis*, and *Escherichia coli*, indicating a polymicrobial infection likely of odontogenic origin.

### 2.4. Intraoral Findings at 4 Years of Age

During the initial dental examination at age four, intraoral swelling was noted in multiple areas. These lesions did not impair feeding but caused concern for caregivers. No pain, fever, or systemic signs were reported. Clinical findings included the following:

- Multiple hemangiomatous lesions located in both maxillary and mandibular regions, predominantly in the upper left posterior quadrant.
- Firm gingival hypertrophy in the upper left quadrant.
- Traumatic avulsion of primary tooth 51 (due to scooter fall).
- Mixed dentition with partial edentulism due to prior surgical extractions; no cases of agenesis were identified.
- Presence of significant subgingival plaque.
- Cervical caries on tooth 83 with enamel loss and exposed dentin.
- Mildly coated tongue.

**Figure 1:**
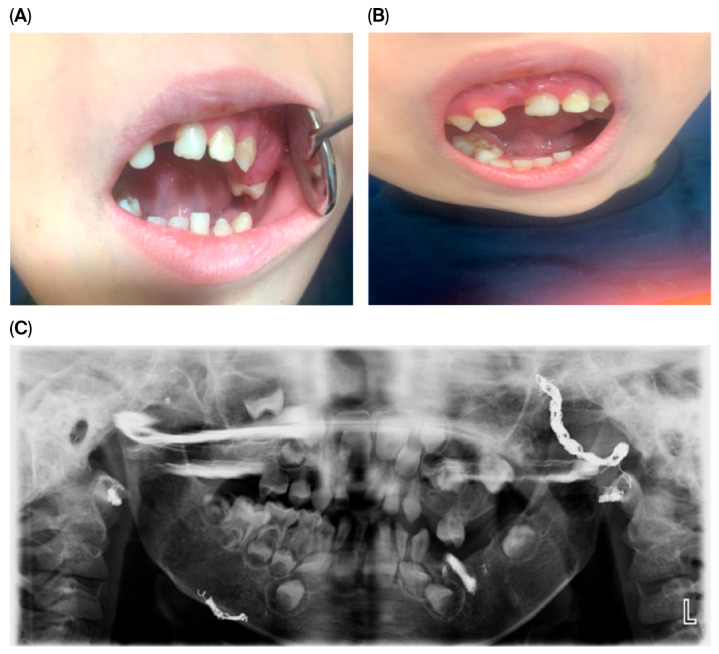
Intraoral and extraoral clinical images at the initial examination (age 4). (**A**) Intraoral view showing gingival overgrowth in the upper molar region. (**B**) Extraoral frontal view demonstrating facial asymmetry and prominent cheeks. (**C**) Panoramic radiograph showing multilocular radiolucent lesions within alveolar bone.

These clinical features are illustrated in Figure 1, showing intraoral and extraoral views as well as radiographic findings.

The panoramic image also indicates potential future dental challenges, such as delayed or ectopic eruption of permanent teeth, malocclusion, and enamel defects. Recognizing these risks early allowed us to plan preventive monitoring and timely interventions. Overall, these clinical and radiographic findings underscore the critical role of routine dental assessments in detecting early manifestations of systemic vascular anomalies, enabling timely diagnosis and multidisciplinary intervention.

**Figure 2:**
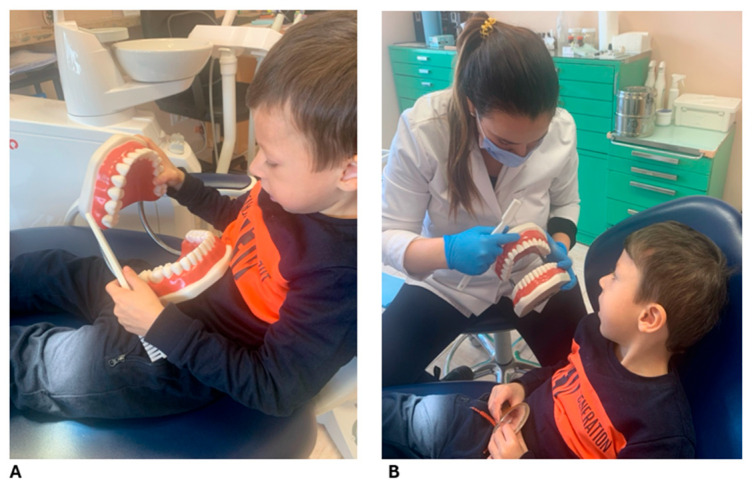
Patient education and motivation for oral hygiene practices at 4 years of age included the following: (**A**) Instruction and motivation for toothbrushing using a manual brush. (**B**) Motivation and demonstration of proper toothbrushing technique with the same manual brush.

Due to the patient’s age and difficulty following plaque disclosure instructions, a fuchsin-based disclosing agent was used to assess oral hygiene, revealing inadequate plaque control with widespread deposits. Despite parental reports of diligent brushing, an excessive fear of provoking bleeding was noted, leading to suboptimal plaque removal. After targeted instruction and demonstration of effective oral hygiene techniques, including age-appropriate motivation and caregiver education (Figure 2), quarterly follow-up visits were scheduled to maintain oral health and mitigate risk factors.

### 2.5. Additional Note

As part of the patient’s overall management, a customized dental care protocol was established in collaboration with oral surgeons and pediatric specialists. Given the vascular fragility and the anatomical extent of the malformation, preventive strategies were prioritized to avoid any invasive interventions. A quarterly recall schedule was implemented for dental check-ups, including plaque control, non-invasive hygiene procedures, and monitoring of the lesion’s stability. All emergency scenarios, including spontaneous bleeding or changes in intraoral tissue, were addressed through a written response plan shared with the caregivers.

### 2.6. Recent Follow-Up (2024–2025)

Management prioritized preventive strategies aimed at avoiding invasive procedures that could trigger uncontrollable hemorrhage or necessitate tooth extractions. Maintaining optimal oral health was emphasized to reduce the risk of complications from untreated carious lesions or infections.

**Figure 3:**
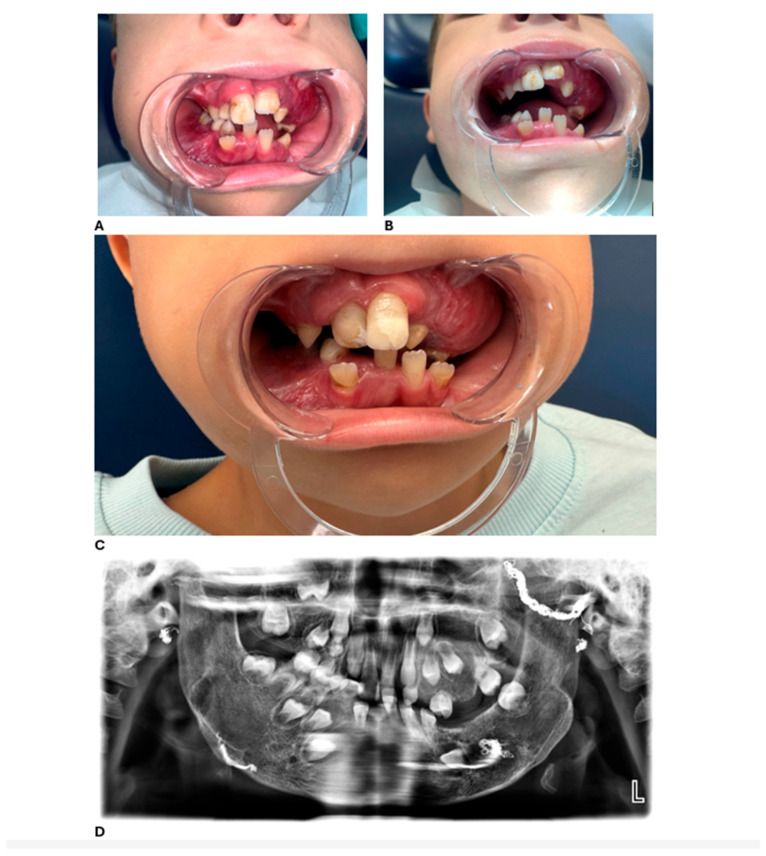
Intraoral findings at 6 and 7 years of age. (**A**) Dysplasia of upper central incisors, ectopic teeth, and gingival overgrowth at 6 years of age. (**B**) Alveolar bone overgrowth in the upper left molar region at 6 years of age. (**C**) Alveolar bone overgrowth, ectopic teeth, and malocclusion at 7 years of age. (**D**) Panoramic radiograph at 7 years of age.

At the most recent dental follow-up, D. has transitioned from full primary dentition to mixed dentition. His permanent maxillary central incisors (teeth 11 and 21) and first molars have erupted, displaying hypoplastic and hypomineralized defects. While these defects are not definitively diagnosed as Turner’s hypoplasia, they are likely attributable to trauma, infection, or previous surgical interventions involving the dental follicles. These teeth were successfully restored with esthetic composite restorations, resulting in significant improvements in both function and appearance. Representative clinical and radiographic findings at ages 6 and 7 are shown in Figure 3.

Based on these findings and the patient’s high risk of caries, a comprehensive preventive and treatment plan was established to guide ongoing dental care.

### 2.7. Preventive and Treatment Plan

Given the patient’s high risk of caries and hypomineralized teeth (11 and 21), a structured preventive and therapeutic plan was implemented:

Preventive measures (to minimize caries and manage bleeding risk):

- Quarterly follow-ups to monitor eruption and detect early lesions.
- Topical fluoride applications and daily use of age-appropriate fluoride toothpaste.
- Fissure sealing of vulnerable teeth.
- Reinforced oral hygiene at home with soft toothbrushes and interdental cleaning under parental supervision.
- Dietary guidance to limit sugary foods and snacks.
- Emergency plan for bleeding/trauma, coordinated with pediatricians/hematologists.

Therapeutic measures (restorative interventions for hypomineralized teeth):

- Minimally invasive restorations for hypomineralized and carious teeth.
- Conservative composite restorations on teeth 11 and 21.
- Regular monitoring of occlusion and dental development to plan any necessary future interventions.
- Interdisciplinary coordination for any surgical needs.

Future considerations (monitoring and adjustment as dentition progresses):

- Adjust preventive and restorative strategies as permanent dentition progresses.
- Continuous monitoring of enamel defects, tooth mobility, and occlusal changes.

Due to the patient’s high hemorrhagic risk, all invasive interventions, including partial prostheses and orthodontic procedures, are currently contraindicated. Preventive care focuses on maintaining a soft, non-traumatic diet, minimizing oral irritation, and closely monitoring dental development. Any future interventions will be carefully evaluated in a multidisciplinary context to balance dental needs with bleeding risk.

These preventive and therapeutic strategies form part of a structured, multidisciplinary approach, supporting the patient and family in navigating care pathways in Bulgaria, where standardized VMOS protocols are yet to be established. Moreover, the findings underscore the long-term dental sequelae associated with VMOS and emphasize the importance of consistent dental monitoring, particularly during transitional dentition stages. This case further highlights the critical role of dental professionals in disease surveillance and restorative care for rare systemic conditions with oral manifestations. Regular monitoring during mixed dentition remains essential for early detection of emerging anomalies and for timely adjustment of preventive strategies aligned with the patient’s evolving dentofacial profile. In this context, our dental team also supported the family in establishing a practical, locally adapted protocol for managing potential bleeding episodes in Bulgaria, where structured care pathways for VMOS are currently lacking.

## 3. Discussion

This case highlights a carefully structured, multidisciplinary management approach that emphasizes preventive care and ongoing monitoring to mitigate the complex risks associated with intraosseous vascular malformations in pediatric patients [1,11].

Primary intraosseous vascular malformations represent exceptionally rare congenital anomalies that often first manifest within the dental setting, underscoring the critical importance of early recognition by oral health professionals. In the present case, a pediatric patient harboring a homozygous ELMO2 mutation with clinical features consistent with VMOS (Vascular Malformation—Osteolytic Subtype) demonstrated the full spectrum of associated dental and systemic complications [12].

Although VMOS remains poorly characterized, with fewer than ten genetically confirmed pediatric cases, to date, the condition consistently features fragile, immature vascular structures within craniofacial bones, particularly the mandible and maxilla. The oral cavity thus serves as a crucial window into early systemic pathology. In our case, gingival hypertrophy, intraoral swelling, and spontaneous bleeding were first noted during a routine dental examination, highlighting the pivotal diagnostic role of the pediatric dentist. These early clinical signs prompted advanced imaging and genetic testing, leading to a timely diagnosis [1,3,10,13].

Clinicians should consider a broad differential diagnosis when evaluating pediatric patients presenting with gingival hypertrophy, intraoral bleeding, jaw expansion, or enamel defects. Potential conditions include other craniofacial vascular anomalies, such as arteriovenous malformations or hemangiomas, genetic syndromes affecting craniofacial development, osteolytic lesions, and inflammatory or infectious processes that may mimic early VMOS manifestations. Recognizing these possibilities highlights the diagnostic value of careful oral examination and the pivotal role of both general and pediatric dentists in initiating timely investigations and coordinating multidisciplinary care [11,14].

Like VMOS, rare genetic disorders such as ectodermal dysplasia (ED) can produce notable dental sequelae. In ED, congenital defects primarily affect tooth number and morphology, often resulting in hypodontia or anodontia, conical teeth, and variable enamel quality. In contrast, VMOS patients typically present with a full complement of teeth, but structural defects such as enamel hypoplasia, hypomineralization, and discoloration are common. Despite these differences, management strategies converge on early recognition, individualized preventive care, and restorative interventions to mitigate functional and esthetic consequences [15,16,17].

From a dental perspective, the challenges posed by VMOS are multifactorial. Spontaneous bleeding and soft tissue overgrowth are commonly observed in patients with ELMO2-associated intraosseous vascular malformations [3]. In addition, the condition significantly disrupts normal tooth development and eruption. Factors contributing to these dental issues may include facial bone expansion, local inflammation, surgical interventions, or trauma. Consequently, tooth mobility, enamel defects such as hypoplasia or hypomineralization, and early curiosity often occur. The management of these complications requires a comprehensive preventive and restorative strategy. This includes esthetic composite restorations as well as reinforced oral hygiene measures [3,10,18].

Compared with previously reported cases of VMOS and other rare craniofacial vascular malformations, this patient demonstrates a unique longitudinal follow-up, integrating preventive dental management, restorative interventions, and monitoring of complications such as cerebral abscesses. While prior reports have mainly focused on systemic features, this case provides a comprehensive view of dental sequelae, underscoring the essential role of pediatric dentists in early recognition, intervention, and long-term care [5,18,19].

Furthermore, the case highlights the necessity of a multidisciplinary approach, involving collaboration among pediatric dentists, oral surgeons, radiologists, and geneticists, to ensure safe and effective management of rare vascular anomalies in children.

When invasive intervention is required—as in this case where a biopsy attempt triggered profuse hemorrhage necessitating coil embolization—the high risk of iatrogenic bleeding becomes starkly apparent. These complications underscore the importance of preoperative vascular imaging, meticulous surgical planning, and interdisciplinary collaboration for safe and effective management. Furthermore, the subsequent development of cerebral abscesses, caused by an odontogenic focus, illustrates the potentially fatal systemic consequences of untreated dental infections in patients with vascular malformations [20,21].

In patients with a history of severe odontogenic infections or systemic complications, such as cerebral abscesses or hemorrhagic cystic lesions, antibiotic prophylaxis should be considered for invasive dental procedures (e.g., extractions, surgical interventions). The recommended regimen for this patient would be amoxicillin 50 mg/kg orally (maximum 2 g) administered 30–60 min prior to the procedure. For children with a penicillin allergy, clindamycin 20 mg/kg orally (maximum 600 mg) can be used as an alternative. Incorporating these preventive measures ensures safer management of high-risk dental procedures in patients with VMOS [21,22,23].

Long-term dental follow-up remains essential. As the child transitioned into mixed dentition, enamel defects affecting the first permanent molars and incisors necessitated early restorative intervention and vigilant monitoring for emerging complications. The patient’s elevated hemorrhagic risk renders future invasive procedures aimed at restoring masticatory function difficult to anticipate and execute. Any such interventions will be planned and critically assessed within a multidisciplinary framework when clinically indicated. In the absence of standardized care protocols, particularly in regions lacking structured pathways for VMOS management, the dental professional may assume a central role not only as a clinician but also as a care coordinator, guiding families through the local healthcare landscape. Additionally, reports from pediatric case series emphasize that individualized management and a multidisciplinary follow-up strategy over several years are critical to optimize outcomes and prevent complications during growth [10,18,24,25].

This case contributes to the growing body of knowledge on VMOS by providing detailed insights into its oral manifestations, clinical progression, and management strategies. Intraosseous arteriovenous malformations in the jaws are rare congenital vascular abnormalities that dentists may encounter and should be suspected in cases of unexplained intraoral bleeding, jaw expansion, or tooth mobility [26]. Given that facial bone hypertrophy, gingival changes, and enamel abnormalities often emerge in early childhood, pediatric dentists play a pivotal role in early recognition, timely imaging, and coordination of care [11]. Even in extremely rare conditions like VMOS, vigilant attention to early oral signs can facilitate prompt diagnosis and multidisciplinary intervention, ultimately improving patient outcomes.

The presence of an *ELMO2*-associated intraosseous vascular malformation introduces significant challenges to routine dental care, particularly in pediatric patients. In our case, we emphasize the importance of a preventive, multidisciplinary approach. Minimally invasive procedures, hospital-based planning for any surgical needs, and close monitoring with regular imaging are essential. These steps align with the current literature on vascular anomalies in the craniofacial region, which underscores the necessity of individualized care plans and interdisciplinary coordination to mitigate hemorrhagic risk and preserve oral health [11,22,26].

## 4. Conclusions

This case of VMOS associated with homozygous ELMO2 mutation highlights the diagnostic and management challenges of rare intraosseous vascular malformations in children. Early recognition of oral signs by pediatric dentists, combined with genetic testing and multidisciplinary care, is essential to prevent serious complications. Preventive dental strategies and cautious, tailored treatment are crucial to maintain oral health and improve patient outcomes. This report contributes to the limited knowledge on VMOS and underscores the pivotal role of dental professionals in managing complex vascular anomalies.

## Data Availability

The data presented in this study are available on request from the corresponding author. The data are not publicly available due to privacy restrictions.

## Abbreviations

The following abbreviations are used in this manuscript:

VMOS	Vascular malformation of the intraosseous subtype
DNA	Deoxyribonucleic acid
EDTA	Ethylenediaminetetraacetic acid
NGS	Next-Generation Sequencing
CT	Computed tomography
MRI	Magnetic resonance imaging
AV	Arterio-venous
